# Co-Occurrence of Moniliformin and Regulated *Fusarium* Toxins in Maize and Wheat Grown in Italy

**DOI:** 10.3390/molecules25102440

**Published:** 2020-05-23

**Authors:** Terenzio Bertuzzi, Paola Giorni, Silvia Rastelli, Patrizia Vaccino, Chiara Lanzanova, Sabrina Locatelli

**Affiliations:** 1 Department of Animal, Food and Nutrition Science—DIANA, Università Cattolica del Sacro Cuore, Via Emilia Parmense 84, 29122 Piacenza, Italy; silvia.rastelli@unicatt.it; 2 Department of Sustainable Crop Production—DIPROVES, Università Cattolica del Sacro Cuore, via Emilia Parmense 84, 29122 Piacenza, Italy; paola.giorni@unicatt.it; 3 Council for Agricultural Research and Economics (CREA)—Research Center for Cereal and Industrial Crops, Strada Statale, 11 per Torino km 2.5, 13100 Vercelli, Italy; patrizia.vaccino@crea.gov.it; 4 Council for Agricultural Research and Economics (CREA)—Research Center for Cereal and Industrial Crops, Via Stezzano, 24, 24126 Bergamo, Italy; chiara.lanzanova@crea.gov.it (C.L.); sabrina.locatelli@crea.gov.it (S.L.)

**Keywords:** moniliformin, *Fusarium* mycotoxins, maize, wheat

## Abstract

The co-occurrence of moniliformin (MON), fumonisins (FBs), and deoxynivalenol (DON) was evaluated in maize, durum, and common wheat grown in different experimental fields located in several Italian regions. MON was quantified using a LC-MS/MS method adding lanthanum ions in the mobile phase. In maize, MON contamination was widespread and considerable; the toxin was detected in almost all the samples (95.1%) and exceeded 500 and 1000 µg kg^−1^ in 42.0% and in 18.5% of samples, respectively. Significant positive correlation was found between MON and FB contamination levels. When there were not droughty climate conditions, a positive significant correlation was found between growing degree days (GDD) and MON values. In wheat, MON contamination was not widespread like in maize and it was lower in common wheat than in durum wheat. In durum wheat, MON was detected in 45.0% of the samples with only 6 samples (7.5%) exceeding 500 µg kg^−1^, while in common wheat the toxin was detected above the LOD in 18.7% of samples exceeding 100 µg kg^−1^ in only two samples (2.5%). No correlation was found with DON contamination. Climate conditions influenced both MON and DON occurrence.

## 1. Introduction

**Figure 1:**
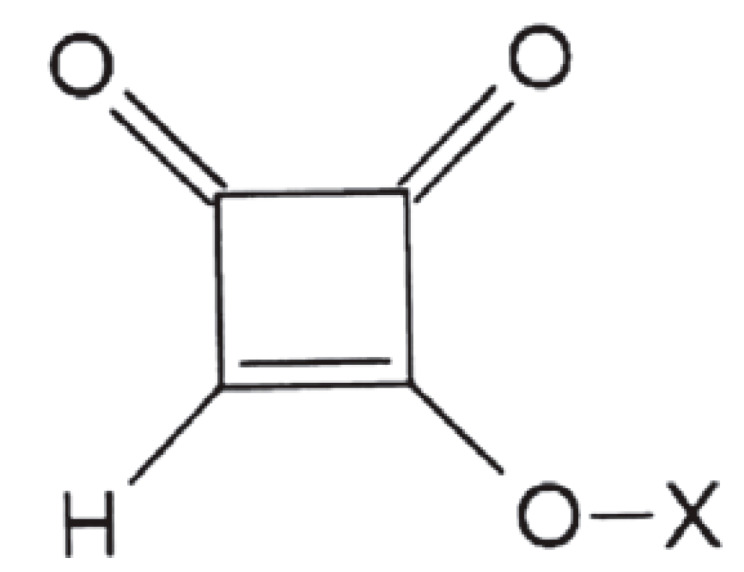
Chemical structure of moniliformin (X=H, Na, or K).

Mycotoxins are one of the most important contaminants in cereals. They are toxic secondary metabolites usually produced, in favorable environmental conditions, by *Aspergillus*, *Penicillium*, *Alternaria*, and *Fusarium* fungi. Among these species, *Fusarium* are the most prevalent mycotoxin-producing fungi in Central and Southern Europe. *Fusarium* species are known as producers of several mycotoxins, including regulated ones fumonisins, trichothecenes, and zearalenone. Generally, in Italy, the most widespread mycotoxins for maize and wheat are fumonisins (FBs) and deoxynivalenol (DON), respectively. Besides these toxins, moniliformin (MON) is a not negligible emerging *Fusarium* mycotoxin occurring in cereals; generally, higher levels have been found in maize than in other crops. MON is mainly produced by *F. subglutinans, F. temperatum, F. verticilloides*, and *F. proliferatum*. Moreover, the last two fungal species are also able to produce FBs [1]. MON is a highly polar and acidic molecule (Figure 1) and occurs as a water-soluble sodium or potassium salt [2]. 

Cardiotoxicity and hepatotoxicity are its major adverse health effects, as indicated by the Panel on Contaminants in the Food Chain (CONTAM) of the European Food Safety Authority (EFSA) [3]. The main target of MON seems to be enzymes with thiamine as a cofactor. Since these enzymes are part of the respiratory chain, any impairment of their activity results in a shortage of energy, respiratory stress, and myocardial loss of functionality. Furthermore, MON can reduce the activity of glutathione peroxidase and glutathione reductase, thus increasing oxidative stress [4]. Concerning sub-acute toxicity in male rats, the relative LD50 has been set at 6 mg kg^−1^ bw per day for mortality and at 15 mg kg^−1^ bw for cardiotoxicity [5]. Due to the low number of studies on MON toxicity, the EFSA CONTAM Panel has not been able to establish a TDI for this toxin. Furthermore, a recent review reported an interactive toxicity of MON and FBs [6]. To date, no regulatory limits have been fixed for MON and the EFSA has stated that further toxicity studies are needed, also recommending the collection of more data on MON occurrence [3]. Although MON can contaminate several cereal commodities, few data are available in literature about its spread and the relation with meteorological conditions, growing season and area has been poorly investigated. Considering the most recent studies, a not negligible MON contamination was found in cereals produced both in Scandinavian countries and in Southern Europe, showing that MON can be produced in different climate conditions and by several *Fusarium* species [7,8,9,10]. As regards the Mediterranean area, considerable MON contamination was detected in Serbian maize produced from 2016–2018 showing values up to 3856 μg kg^−1^ [11]; in Italy MON was found in 75% of 12 naturally contaminated winter wheat samples (maximum value 80 μg kg^−1^) [12] and in almost all 108 maize samples grown in Northwestern Italy (maximum value 2606 μg kg^−1^) [13]. Beccari et al. (2020) [14] showed that durum wheat cultivated in Central and Northern Italy may be subject to *Fusarium* mycotoxin accumulation, including MON; in our recent study regarding the development of an alternative LC-MS/MS method for its determination, MON was detected in 10/10 maize and in 5/10 wheat samples [15]. Finally, Scarpino et al. (2020) [16] reported a weak decrease of MON contamination after different large-scale maize dry-milling processes. In this work, MON occurrence in maize and wheat (common and durum) grown in fields located in different Italian regions was investigated and possible correlation with FB and DON contamination and with climatic conditions were evaluated.

This study took into consideration the occurrence of moniliformin, an emerging mycotoxin, in the principal cereals cultivated in Italy—maize and wheat—and its correlation with the most common *Fusarium* mycotoxins, fumonisins and deoxynivalenol. Furthermore, influence of climate conditions during the growing period of these cereals was evaluated.

## 2. Results and Discussion

### 2.1. Maize

Grain moisture at harvest ranged from 22.2% to 26.0%. Descriptive statistics (incidence, mean, median and range) of the results obtained for MON, FBs, and DON contamination are reported in Table 1.

**Figure 2:**
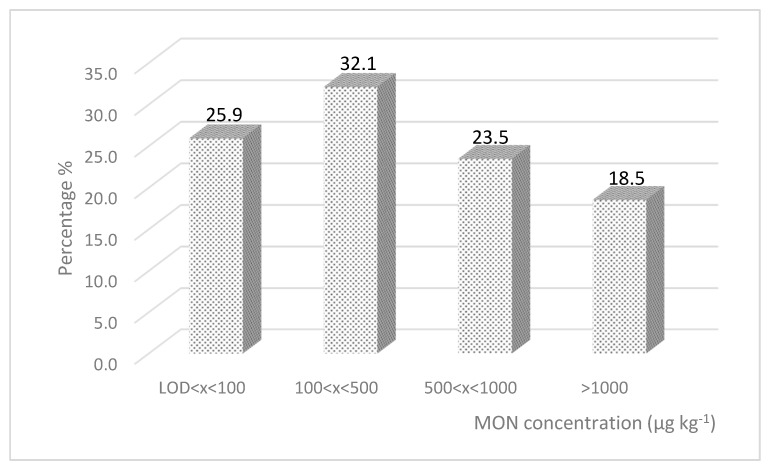
MON relative frequency distribution in maize samples collected in Northern Italy in 2018.

The MON contamination was very widespread and remarkable; the toxin was detected in almost all the samples (95.1%) and exceeded 500 and 1000 µg kg^−1^ in 42.0% and in 18.5% of samples, respectively. Figure 2 shows the MON relative frequency distribution in maize samples. 

This high incidence confirmed the results of previous surveys carried out in Italy and in neighboring countries of the Mediterranean area. Scarpino et al. [13] reported MON contamination in 93% of maize samples collected in Northwestern Italy between 2008 and 2011 (maximum value 2606 µg kg^−1^); in Serbian maize, MON was often detected from 2012–2015 (maximum level 8710 µg kg^−1^) [17] and from 2016–2018 (maximum level 3856 µg kg^−1^) [11]. Finally, a widespread occurrence was found in maize grown in Kosovo from 2009–2010 (maximum levels 320 µg kg^−1^) [18]. Significantly different contamination was found in the nine locations considered (*p* < 0.01). In particular, in Bergamo (Field 3), both mean and median values were close to 2000 µg kg^−1^. On the other hand, no differences were found among the hybrids. The different MON contamination levels in the fields was probably due to the different climate conditions. In particular, the field with the highest level of contamination showed the highest growing degree days (GDD) value (2557 °C) and considerable total rainfall (649 mm); for the other fields, GDD value was always lower than 2250 °C, while rainfall ranged between 313 and 943 mm. These data confirmed that MON production was favored to a great extent by temperature, but not dry climate conditions; Scarpino et al. [19] reported low MON contamination levels when less rainfall occurred. Moreover, Jajic et al. [11] affirmed that warm weather associated with high humidity enabled *Fusarium* infection of the ears and consequently high MON contamination. In our study, both GDD and rainfall were remarkably higher than those reported by Scarpino; considering that 2018 was characterized by warm but not droughty climate conditions in Northern Italy, a positive significant correlation was found between GDDs and MON values (α = 0.05).

As regards contamination with regulated *Fusarium* mycotoxins, FBs were found in almost all the samples (92.6 %), sometimes at very high levels (maximum value 43,297 µg kg^−1^). However, ANOVA showed significant differences (*p* ≤ 0.01) only for Field 2 located in Chivasso that was less contaminated by FBs (median value 13 µg kg^−1^), while all the other fields did not show statistically significant differences for FBs contamination, with median values from 1100 to 6610 µg kg^−1^. Positive significant correlation was found between FBs and GDDs (α = 0.05) while a negative significant correlation was found between FBs and total rainfall (α = 0.05). This confirmed previous studies in which fumonisin production in maize grains resulted as being influenced largely by temperature and less by rainfall [20,21].

DON contamination was widespread (80.2% of positive samples) but at very low levels (only three samples exceeded 500 µg kg^−1^). Significant differences (*p* ≤ 0.05) were found between the locations, with Field 1 (Castagnole) as the most contaminated and Field 2 (Chivasso) the least. No significant correlations were found between DON and meteorological parameters.

**Figure 3:**
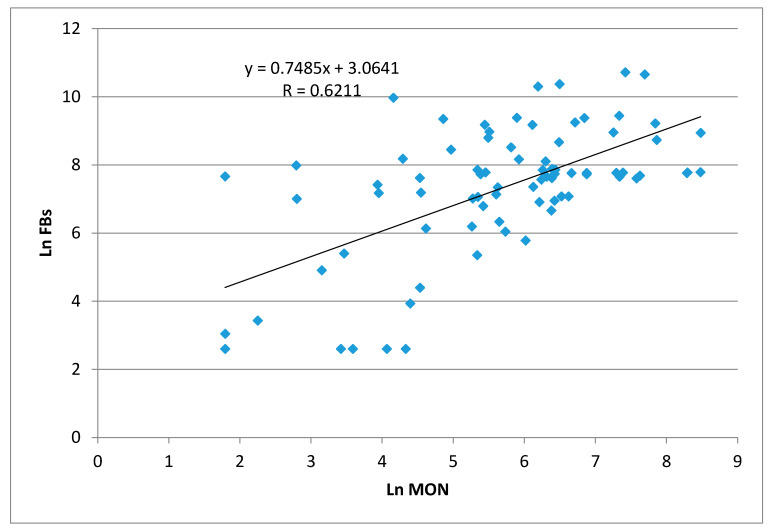
Correlation between moniliformin (MON) and fumonisins (FBs) in maize samples. Data were ln transformed.

Both MON and FBs can be produced by *F. proliferatum* and *F. verticilloides*, as previously reported [22]; therefore, the relationship between their concentration levels was examined. Significant positive correlation (α = 0.05) was found between these mycotoxins (Figure 3), confirming the findings reported by Scarpino et al. [19], which showed a significant correlation in maize grown in the same field in the years 2008–2010. These results showed that FB contamination can often be associated with MON occurrence in maize produced in Northern Italy and that *F. proliferatum* and *F. verticilloides* can be considered the main fungal species responsible for their production. Recently, a review investigated the combined effects of MON and co-occurring *Fusarium* mycotoxins in feed on farm animals; even if a small number of studies are present in literature, an interactive toxicity between MON and FBs was reported [6].

### 2.2. Durum Wheat

**Figure 4:**
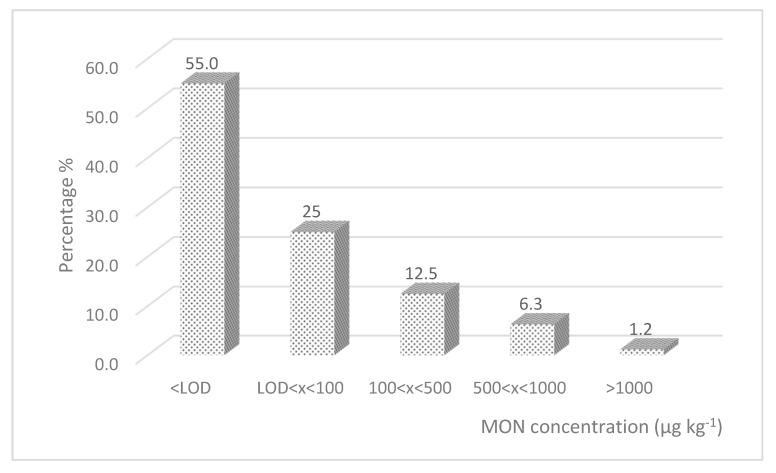
MON relative frequency distribution in durum wheat samples collected in Italy in 2019.

Kernel moisture at harvest was similar, ranging from 10.1% to 12.6%. Descriptive statistics (incidence, mean, median, and range) of the results obtained for MON and DON contamination are reported in Table 2. MON contamination in durum wheat was not as widespread as for maize (45.0% of positive samples) and only six samples, corresponding to 7.5%, exceeded 500 µg kg^−1^ (Figure 4). Nevertheless, contamination was not negligible and, out of eight experimental fields, only the one in Sicily was completely free of contamination. MON occurrence in Italian durum wheat has been scarcely investigated. Recently, Beccari et al. [14] reported contamination with different *Fusarium* mycotoxins in durum wheat collected in three different Italian regions (Emilia-Romagna, Umbria, and Sardinia); MON was detected at not negligible levels (maximum 610 µg kg^−1^) in samples from Northern (Emilia-Romagna) and Central (Umbria) Italy, with significant differences (Northern > Central Italy). In our study, the fields were located in Central and Southern Italy and MON was detected at values higher than 100 µg kg^−1^ only in three fields of Central Italy. 

Of these, only one, Field 2 (Barbaruta), with mean and median values close to 500 µg kg^−1^, showed a significantly higher contamination (*p* < 0.01). No differences were found between the other locations or durum wheat varieties. 

In Italy, the main *Fusarium* mycotoxin found in wheat is generally DON with durum wheat often more heavily contaminated than common wheat [23]. In several countries, FBs were generally detected at low levels in wheat and derived products [24]; also, in Italy the contamination levels were low [25]. Otherwise, DON was found in Italian wheat at not negligible levels. In our study, DON was detected in almost all durum wheat samples (96.2%), but only in 10 samples (12.5%) did the toxin exceed 300 µg kg^−1^, never reaching 500 µg kg^−1^. These data confirmed the findings of previous studies which reported low DON contamination in wheat cultivated in Central and Southern Italy [26]. Only in one location (Rieti, Field 1), with a mean value close to 300 µg kg^−1^, was DON significantly higher (*p* < 0.01). The comparison between MON and DON levels showed that, in 33.7% of samples, MON was higher than DON contamination. Their co-occurrence was found in 43.7% of samples and no significant correlation was obtained, speculating that these toxins were produced by different *Fusarium* species. This result confirmed the findings of Beccari et al. [27], which identified the different *Fusarium* species occurring in Italian durum wheat and evaluated their mycotoxigenic potential in vitro. In particular, *F. avenaceum* strains were characterized by high MON production, while *F. graminearum* strains were in prevalence DON producers.

In Italy, wheat is cultivated during the winter-spring period; considering the different weather conditions registered for each field, MON production seemed to increase in cases of GDD levels higher than 2800 °C and moderate rainfall, and DON contamination increase with low GDDs and high rainfall. A significant negative correlation was found between DON and GDD values, while a significant positive correlation between DON and rainfall (α = 0.05). In particular, high DON levels were determined in wheat grown in fields where high rainfall (close to 100 mm, data not shown), associated with low GDDs (lower than 350 °C), occurred in April (before and during flowering). Actually, the important role of rainfall in DON infection of wheat has already been underlined in other studies, where a decrease in rainfall prior to flowering was found to represent the key factor for lower DON contamination [28]. Interestingly, MON behavior was found to be exactly the opposite with a significant positive correlation with GDDs and a significant negative correlation with rainfall (α = 0.05).

### 2.3. Common Wheat

Kernel moisture at harvest ranged from 10.2% to 12.8%. Descriptive statistics (incidence, mean, median, and range) of the results obtained for MON and DON contamination are reported in Table 3.

MON occurrence was very limited; the toxin was detected above the LOQ in 13.7% of samples (18.7% above the LOD) and only two samples exceeded the value of 100 µg kg^−1^. Interestingly, in the five fields considered for sampling, two of them (one located in Northern—Conselice, Field 3, and one in Southern Italy—Foggia, Field 5) did not show MON contamination. DON contamination was significantly different (*p* ≤ 0.01) between the fields: absent or scarce in fields 4 and 5 (Southern and Central Italy), very high in Field 1 and 2, both in Northern Italy (Cigliano and S.Angelo Lodigiano), where 20 out of 32 samples (62.5%) exceeded the EU legal limit of 1250 µg kg^−1^ for food consumption. In the latter two fields, MON was rarely present (28%) and lower than 100 µg kg^−1^. The statistical results (Table 3) were obtained using Tukey post-doc test, like for other cereals; however, the *p* value, determined using Levene test, was 0.049, very close to the 0.05 value to accept means as different. Then, the Games–Howell test was used and the results were slightly different: Field 1 = Field 2; Field 2 = Field 1 and Field 4; Field 1 ≠ Field 4; Field 3 = Field 5. As for durum wheat, MON occurrence has been scarcely investigated in Italian common wheat; from these results, it seems that in Italy MON contamination in common wheat is less of a problem than for durum wheat. Both MON and DON were positively correlated with rainfall (α = 0.05), while a negative significant correlation was found between DON and GDD levels (α = 0.05). Unlike durum wheat, it appears notable that high DON levels were determined for Field 1 and 2, when high rainfall (129 and 116 mm, data not shown) occurred in April was associated with GDD values (385 and 390 °C) higher if compared with other fields.

## 3. Materials and Methods

### 3.1. Samples

A total of 241 samples of maize (*n* = 81), durum wheat (*n* = 80), and common wheat (*n* = 80) were analyzed. During the 2018 season, maize was collected at harvest from nine experimental fields located in the main Italian maize producing regions (Piedmont, Lombardy, Veneto, Friuli V.G., Emilia-Romagna); seven different hybrids were analyzed for each field (FAO class from 500 to 700). At harvest maize ears were collected by hand and dried at 40 °C for a week; for each field, 12 ears of each hybrids (about 4 kg) were collected. The ears were shelled using an electric sheller, and kernels from each plot were mixed thoroughly in order to obtain a random distribution. Samples (1.5 kg) were taken and grains were milled with a ZM 200 Retsch Ultra-Centrifugal mill equipped with a DR 100 vibratory feeder (Retsch GmbH, Haan, Germany) to a 1 mm sieve size and stored at 4 °C until the time of analysis.

Durum (10 varieties) and common (16 varieties) wheat were collected during the 2018–2019 growing season from eight and five fields, respectively, located in several Italian regions (Piedmont, Emilia-Romagna, Tuscany, Latium, Apulia, Sicily). From each field, three 2 kg grain samples for each variety were collected and mixed accurately. A subsample of 400 g was then milled as described previously, and stored at 4 °C until the time of analysis. Durum wheat sampling was organized with the support of the following research centers: CREA—Cereal and Industrial Crops (Acireale, CT); and CREA—Engineering and Agro-Food Processing (Roma).

### 3.2. Meteorological Data

Meteorological daily data were collected from ARPA (Regional Agency for Environmental Protection) stations, located near the experimental fields, in the period 1 April–15 October 2018 for maize and 1 October 2018–30 June 2019 for wheat. The collected meteorological parameters were: mean daily temperature (T) and daily rainfall (mm). Data were used to calculate growing degree days (GDD) and total rainfall occurred during the growing season. The growing degree days (GDD) were calculated according to McMaster and Wilhelm [29] formula

GDD = [(T_max_ + T_min_)/2 − T_base_] (1)

where T_max_ is the daily maximum air temperature, T_min_ is the daily minimum air temperature, and T_base_ is the temperature below which the process of interest does not progress. For maize and wheat, T_base_ is 8.5 °C 0.5 °C respectively (Tables S1–S3).

### 3.3. Reagents and Standards

Chemicals and solvents used for extraction and clean-up were ACS grade or equivalent (Carlo Erba, Milan, Italy); deionized water was purified through a Milli-Q treatment system (Millipore, Bedford, MA, USA). For LC-MS/MS analysis, water, methanol, and acetonitrile were HPLC grade (Merck, Darmstadt, Germany). MON (as sodium salt), Lanthanum (III) chloride heptahydrate (LaCl_3_·7H_2_O), were obtained from Sigma-Aldrich (St. Louis, MO, USA). A MON stock standard solution was prepared in acetonitrile at a concentration of 100 mg L^−1^; working solutions were obtained by dilution using water:methanol 15 + 85 *v*/*v*. All the solutions were stored at −20 °C when not in use.

### 3.4. LC-MS/MS Analysis for Moniliformin Determination

MON was quantified using the method of Bertuzzi et al. [15]. Briefly, after extraction from cereal samples (10 g each) with 40 mL of a mixture acetonitrile: water 50 + 50 *v*/*v* for 60 min, filtration on a folded filter paper and dilution (1 + 1) with methanol:water 85 + 15 *v*/*v*, the extract was injected into the LC-MS/MS system. The HPLC-MS/MS system consisted of a LC 1.4 Surveyor pump (Thermo Fisher Scientific, San Jose, CA, USA), a PAL 1.3.1 sampling system (CTC Analytics AG, Zwingen, Switzerland) and a Quantum Discovery Max triple quadrupole mass spectrometer; the system was controlled by an Excalibur 1.4 software (Thermo Fisher Scientific, San Jose, CA, USA). MON was separated on a Supelcosil LC-NH_2_ column (75 × 3 mm, 3 µm, Supelco, Bellefonte, PA, USA) using a gradient elution with 25 mM ammonium acetate containing 1.25 mM LaCl_3_·7H_2_O, and methanol as mobile phase A and B, respectively. The gradient program was a linear gradient from 10% to 30% of solvent A in 3 min, then isocratic for 1 min; column conditioning lasted 7 min. The flow rate was 0.3 mL min^−1^. The ionization was carried out with an ESI interface (Thermo-Fisher) in negative mode as follows: spray capillary voltage was 3.5 kV, sheath and auxiliary gas 40 and 15 psi, respectively; skimmer 9 V, temperature of the heated capillary 350 °C. The mass spectrometric analysis was performed in selected reaction monitoring (SRM). For fragmentation of the [M − H]^−^ ion (97 *m*/*z*), the argon collision pressure was set to 1.2 mTorr and the collision energy to 21 V. The detected and quantified fragment ion was 41 *m*/*z*. Quantitative determination was performed by an LC-Quan 2.0 software (Thermo-Fisher, Waltham, MA, USA). The limit of detection (LOD) and of quantification (LOQ) were 10 and 25 µg kg^−1^, respectively.

### 3.5. Analysis for Fumonisin and Deoxynivalenol Determination

FB content was determined only in maize, DON content both in maize and wheat. For the extraction, 25 mL of methanol:water 70 + 30 *v*/*v* and 25 mL of water for FBs and DON, respectively, was added to 5 g of ground kernel sample. The mixtures were then shaken vigorously for 3 min in a shaker and the extracts were filtered through a Whatman no. 1 filter. Mycotoxin concentration levels were determined by the Enzyme-Linked Immunoassorbent Assay (ELISA). The sample extracts were analyzed using the Ridascreen FBs (R-Biopharm R3401) and the Ridascreen DON (R-Biopharm R5906) ELISA test kit, for total fumonisin (FB1, FB2, and FB3) and DON content, respectively. The Ridascreen^®^ R-Biopharm kit tests were performed using the Chemwell Automatic Awareness Engineer (inc.). Mycotoxin extraction and analysis were performed according to the manufacturer’s instructions.

The limit of detection (LOD) was 25 and 18 µg kg^−1^, for FBs and DON, respectively. In order to confirm the data, 25 samples for each cereal were also re-analyzed using LC-MS/MS and GC-MS methods developed in our laboratory [23,30] for FB and DON analysis, respectively (Table S4). The LOD was 10 µg kg^−1^ for FBs and 5 µg kg^−1^ for DON.

### 3.6. Statistical Analysis

Analysis of variance (ANOVA) was calculated using the statistical package IBM SPSS statistics 21 (IBM Corp., Armonk, NY, USA). Significant differences were highlighted using the Tukey test (*P* ≥ 95%). The data of mycotoxin contaminations were ln transformed before statistical analysis [31]. Data correlation was evaluated by Pearson’s correlation test (*P* ≥ 95%).

## 4. Conclusions

The object of this work was based on the EFSA recommendation about the collection of more data on MON occurrence. Maize grown in Northern Italy is often contaminated with this mycotoxin; levels of contamination are influenced by the location of fields and probably by their climate conditions. MON occurrence is statistically correlated with FB contamination, suggesting that *Fusarium* species are able to produce both mycotoxins, when favorable meteorological conditions occur. The good agronomic practices developed to decrease the risk of FB contamination should also be tested for a MON reduction. These results confirmed the widespread occurrence found in some neighboring countries of the Mediterranean area; however, more data on its occurrence in maize cultivated in several European countries could be useful to better evaluate the agronomic and climate conditions favorable to its production. Confirming previous studies, lower levels were found in wheat than in maize; however, MON occurrence was not negligible, especially for durum wheat. For this cereal, the main Italian producing areas were considered (Central and Southern Italy) and the results showed a different trend between these two areas. Durum wheat is also cultivated in some regions of Northern Italy and following studies could afford to obtain an overall evaluation of MON occurrence in this cereal grown in Italy. Finally, common wheat showed a limited MON contamination, even in samples with high DON occurrence, speculating, as already reported, that the *Fusarium* producing species are different.

In conclusion, this work showed that this emerging mycotoxin can often occur in cereals; therefore, further studies are required to obtain more information about its toxicity and, successively, to evaluate the introduction of maximum recommended limits.

## Figures and Tables

**Table 1:** Descriptive statistics of moniliformin (MON), fumonisins (FBs), and deoxynivalenol (DON) contamination (μg kg^−1^) for maize samples collected in Northern Italy in 2018.

	MON	FBs	DON
*Field 1 (Castagnole, TO, Piedmont)*			
Positives (%)	100	100	100
Mean ± std. dev.	408 ± 234 ^A,B^	1491 ± 815 ^A^	149 ± 180 ^a^
Median	372	1180	103
Range	93–751	780–3510	44–614
*Field 2 (Chivasso, TO, Piedmont)*			
Positives (%)	78	33	44
Mean ± std. dev.	71 ± 94 ^C^	121 ± 185 ^B^	<LOD ^c^
Median	35	<LOD	<LOD
Range	<LOD-307	<LOD-420	<LOD
*Field 3 (Bergamo, BG, Lombardy)*			
Positives (%)	100	100	78
Mean ± std. dev.	2255 ± 1610 ^A^	2227 ± 157 ^A^	28.6 ± 70.3 ^b,c^
Median	1953	2290	<LOD
Range	592–4800	2000–2400	<LOD-216
*Field 4 (Fontanella, BG, Lombardy)*			
Positives (%)	89	100	78
Mean ± std. dev.	592 ± 494 ^A,B^	3296 ± 3542 ^A^	42 ± 87 ^a,b,c^
Median	543	2260	<LOD
Range	<LOD-1529	1000–12,600	<LOD-273
*Field 5 (S. Angelo Lod., LO, Lombardy)*			
Positives (%)	100	100	89
Mean ± std. dev.	1231 ± 1550 ^A,B^	6803 ± 3327 ^A^	397 ± 780 ^a,b^
Median	622	6610	<LOD
Range	143–4811	2360–11,890	<LOD-2080
*Field 6 (Arquà Polesine, RO, Veneto)*			
Positives (%)	100	100	89
Mean ± std. dev.	487 ± 545 ^B,C^	5492 ± 4850 ^A^	18 ± 26 ^b,c^
Median	245	2930	<LOD
Range	8–1613	30–11,800	<LOD-83
*Field 7 (S. Bellino, RO, Veneto)*			
Positives (%)	100	100	100
Mean ± std. dev.	135 ± 98 ^B,C^	3383 ± 6797 ^A^	35 ± 89 ^b,c^
Median	92	1100	<LOD
Range	15–283	50–21,320	<LOD-271
*Field 8 (Pozzuolo, UD, Friuli VG)*			
Positives (%)	89	100	78
Mean ± std. dev.	633 ± 853 ^B,C^	3547 ± 3474 ^A^	<LOD ^b,c^
Median	276	1540	<LOD
Range	<LOD-2593	25–9650	<LOD-29
*Field 9 (Mirandola, MO, Emilia-Romagna)*			
Positives	100	100	67
Mean ± std. dev.	777 ± 680 ^A,B^	17,458 ± 18,880 ^A^	<LOD ^b,c^
Median	488	4981	<LOD
Range	192–2190	323–43,297	<LOD-38

^A,B,C^: Values marked by different capital letters in a column are significantly different (*p* ≤ 0.01). ^a,b,c^: Values marked by different lowercase letters in a column are significantly different (*p* ≤ 0.05).

**Table 2:** Descriptive statistics of moniliformin (MON) and deoxynivalenol (DON) contamination (μg kg^−1^) for durum wheat samples collected in Italy in 2019.

	MON	DON
*Field 1 (Rieti, RI, Latium)*		
Positives (%)	20	100
Mean ± std. dev.	6.7 ± 4.6 ^B,C^	305 ± 155 ^A^
Median	<LOD	297
Range	<LOD-18	123–498
*Field 2 (Barbaruta, GR, Tuscany)*		
Positives (%)	80	100
Mean ± std. dev.	519 ± 438 ^A^	222 ± 156 ^A,B^
Median	423	224
Range	<LOD-1500	18–476
*Field 3 (Montelibretti, ROME, Latium)*		
Positives (%)	50	100
Mean ± std. dev.	83 ± 104 ^A,B,C^	83 ± 88 ^B,C,D^
Median	44	49
Range	<LOD-291	<LOD-291
*Field 4 (Marciano, AR, Tuscany)*		
Positives (%)	40	90
Mean ± std. dev.	14 ± 23 ^B,C^	160 ± 129 ^A,B,C^
Median	<LOD	136
Range	<LOD-78	<LOD-408
*Field 5 (Viterbo, VT, Latium)*		
Positives (%)	70	90
Mean ± std. dev.	23 ± 26 ^B,C^	<LOD ^E^
Median	8	<LOD
Range	<LOD-132	<LOD-27
*Field 6 (Tarquinia, VT, Latium)*		
Positives (%)	40	100
Mean ± std. dev.	27 ± 41 ^B,C^	32 ± 33 ^C,D,E^
Median	<LOD	22
Range	<LOD-132	<LOD-101
*Field 7 (Alberese, GR, Tuscany)*		
Positives (%)	60	90
Mean ± std. dev.	196 ± 320 ^A,B^	38 ± 71 ^D,E^
Median	12	<LOD
Range	<LOD-951	<LOD-236
*Field 8 (Libertinia, CT, Sicily)*		
Positives (%)	0	100
Mean ± std. dev.	<LOD ^C^	111 ± 42 ^A,B^
Median	<LOD	112
Range	<LOD	43–181

^A,B,C,D,E^: Values marked by different capital letters within a column are significantly different (*p* ≤ 0.01).

**Table 3:** Descriptive statistics of moniliformin (MON) and deoxynivalenol (DON) contamination (μg kg^−1^) for common wheat samples collected in Italy in 2019.

	MON	DON
*Field 1 (Cigliano, VC, Piedmont)*		
Positives (%)	31.2	93.7
Mean ± std. dev.	15 ± 17 ^A,B^	1740 ± 1398 ^A^
Median	<LOD	1714
Range	<LOD-51	<LOD-4200
*Field 2 (S. Angelo Lod., LO, Lombardy)*		
Positives (%)	25	100
Mean ± std. dev.	17 ± 28 ^A,B^	1445 ± 787 ^A^
Median	<LOD	1333
Range	<LOD-90	342–2600
*Field 3 (Conselice, RA, Emilia-Romagna)*		
Positives (%)	0	43.7
Mean ± std. dev.	<LOD ^B^	90 ± 140 ^B^
Median	<LOD	<LOD
Range	<LOD	<LOD-452
*Field 4 (Barbaruta, GR, Tuscany)*		
Positives (%)	37.5	50
Mean ± std. dev.	41 ± 87 ^A^	96 ± 125 ^B^
Median	<LOD	43
Range	<LOD-349	<LOD-412
*Field 5 (Foggia, FG, Apulia)*		
Positives (%)	0	0
Mean ± std. dev.	<LOD ^B^	<LOD ^C^
Median	<LOD	<LOD
Range	<LOD	<LOD

^A,B,C^: Values marked by different capital letters in a column are significantly different (*p* ≤ 0.01).

## Supplementary Materials

The following are available online, Table S1: Total rainfall (mm), and Growing Degree Days (°C) measured near the experimental field from 1 April to 15 October (for maize). Table S2: Total rainfall (mm), and Growing Degree Days (°C) measured near the experimental field from 1 October to 30 June (for durum wheat). Table S3. Total rainfall (mm), and Growing Degree Days (°C) measured near the experimental field from 1 October to 30 June (for common wheat). Table S4. Comparison between FB and DON results obtained using Elisa technique and chromatographic analysis.
