# Supplementary material for: Co-Occurrence of Moniliformin and Regulated Fusarium Toxins in Maize and Wheat Grown in Italy
Source: Molecules. 2020 May 23;25(10):2440. doi: 10.3390/molecules25102440 (PMC7287609; doi:10.3390/molecules25102440)
Supplement: Supplementary file 1 [file molecules-25-02440-s001.pdf]

Supplementary.

Table S1. Total rainfall (mm), and Growing Degree Days (°C) measured near the experimental field from 1 April to 15 October (for maize).

| | Rainfall (mm) | GDDs (°C) |
|---|---|---|
| *Field 1(To)* | 624 | 2049 |
| *Field 2 (To)* | 943 | 2048 |
| *Field 3 (Bg)* | 649 | 2557 |
| *Field 4(Bg)* | 627 | 2250 |
| *Field 5(Lo)* | 496 | 2224 |
| *Field 6 (Ro)* | 313 | 2183 |
| *Field 7(Ro)* | 449 | 2183 |
| *Field 8(Ud)* | 431 | 2132 |
| *Field 9(Mo)* | 401 | 2204 |

GDDs, Growing Degree Days. °Accumulated growing degree days during period 1 April 2018- 30 October 2018 using a 8,5°C base. Source: ARPA Regional Agency for Environmental Protection of Lombardy, Piedmont, Veneto, Friuli VG, Emilia Romagna).

Table S2. Total rainfall (mm), and Growing Degree Days (°C) measured near the experimental field from 1 October to 30 June (for durum wheat).

| | Rainfall (mm) | GDDs (°C) |
|---|---|---|
| *Field 1(Ri))* | 938 | 2417 |
| *Field 2 (Gr)* | 466 | 3253 |
| *Field 3 (Rome)* | 708 | 3195 |
| *Field 4(Ar)* | 536 | 2869 |
| *Field 5(Vt)* | 556 | 2937 |
| *Field 6 (Vt)* | 589 | 3612 |
| *Field 7(Gr)* | 494 | 3537 |
| *Field 8(Ct)* | 448 | 3775 |

GDDs, growing degree days. °Accumulated growing degree days during period 1 October 2018-30 June 2019 using a 0,5°C base. Source: ARPA Regional Agency for Environmental Protection of Latium, Tuscany, Sicily).

Table S3. Total rainfall (mm), and Growing Degree Days (°C) measured near the experimental field from 1 October to 30 June (for common wheat).

| | Rainfall (mm) | GDDs (°C) |
|---|---|---|
| *Field 1(Vc)* | 521 | 2792 |
| *Field 2 (Lo)* | 502 | 2946 |
| *Field 3 (Mo)* | 435 | 2786 |
| *Field 4(Gr)* | 466 | 3253 |
| *Field 5(Fg)* | 302 | 3777 |

GDDs, growing degree days. °Accumulated growing degree days during period 1 October 2018-30 June 2019 using a 0,5°C base. Source: ARPA Regional Agency for Environmental Protection of Piedmont, Lombardy, Emilia Romagna, Tuscany e Apulia.

Table S4. Comparison between FB and DON results obtained using Elisa technique and chromatographic analysis.

| FBs | | DON | |
|---|---|---|---|
| Elisa | LC-MS/MS | Elisa | GC-MS |
| 1180 | 1076 | 103 | 87 |
| 3510 | 3408 | 614 | 522 |
| 1930 | 1815 | 4 | <LOD |
| 420 | 343 | <LOD | <LOD |
| <LOD | <LOD | 1399 | 1250 |
| <LOD | <LOD | 181 | 140 |
| 2380 | 2166 | 153 | 119 |
| 2020 | 1858 | 236 | 184 |
| 1000 | 922 | 7 | <LOD |
| 2120 | 1931 | 4 | <LOD |
| 1660 | 1460 | <LOD | <LOD |
| 6610 | 6288 | 73 | 55 |
| 4660 | 4474 | 247 | 203 |
| 11890 | 10925 | 408 | 356 |
| 7870 | 7459 | 88 | 74 |
| 11800 | 10858 | 1773 | 1608 |
| 2930 | 2803 | 4200 | 3940 |
| 2570 | 2410 | 4085 | 3851 |
| 21320 | 21015 | 1741 | 1591 |
| 210 | 188 | <LOD | <LOD |
| 1040 | 916 | 412 | 357 |
| 6190 | 5895 | 304 | 274 |
| 3560 | 3258 | 86 | 61 |
| 488 | 396 | 4152 | 3820 |
| 29633 | 28551 | 2630 | 2385 |
